# Development of a Robust UPLC Method for Simultaneous Determination of a Novel Combination of Sofosbuvir and Daclatasvir in Human Plasma:* Clinical Application to Therapeutic Drug Monitoring*

**DOI:** 10.1155/2018/6535816

**Published:** 2018-10-21

**Authors:** Naser F. Al-Tannak, Ahmed Hemdan, Maya S. Eissa

**Affiliations:** ^1^Department of Pharmaceutical Chemistry, Faculty of Pharmacy, Kuwait University, AlJabriyah, Kuwait; ^2^Department of Pharmaceutical Analytical Chemistry, Faculty of Pharmacy, Ahram Canadian University, Giza, Egypt; ^3^Institute of Clinical Chemistry, University Medical Center Hamburg-Eppendorf (UKE), Martinistraße 52, 20246 Hamburg, Germany; ^4^Department of Pharmaceutical Analytical Chemistry, Faculty of Pharmacy, Egyptian Russian University, Cairo, Egypt

## Abstract

A rapid and selective UPLC-DAD method was developed and validated for simultaneous analysis of the novel two-drug combination* Darvoni*® for the treatment of HCV: Sofosbuvir (SF)/Daclatasvir (DC) in human plasma using Ledipasvir as internal standard (IS) where the extraction process was conducted using automated SPE. Although the analysis of the combination after concomitant oral intake of two tablets of SF and DC individually was reported in literature, yet simultaneous analysis of this new combination in human plasma after a single oral dose was not previously reported. The adopted chromatographic separation was achieved on Waters® Acquity UPLC BEH C_18_ column (2.1 × 50 mm, 1.7 *µ*m) as a stationary phase using isocratic elution using a mobile phase system of ammonium formate (pH 3.5; 5 mM) and acetonitrile (60:40 v/v) pumped at a flow rate of 0.2 mL.min^−1^. The UV detection was carried out at 261 nm for SF and 318 nm for DC and IS. SF was eluted at 1.123 min while DC was eluted at 3.179 min. The proposed chromatographic method was validated in accordance with guidelines of FDA for bioanalytical method validation. A linear range was achieved in the range of 25-6400 and 50-12800 ng.mL^−1^ for SF and DC, respectively. The proposed UPLC-DAD method was found to be accurate with % bias ranging between -10.0-7.2 for SF and -6.9-8.0 for DC. Also it was proved to be precise with % CV for intraday precision ranging between 3.8-9.6 for SF and 2.8-9.2 for DC whereas interday precision ranged between 5.1-9.3 for SF and 3.7-9.1 for DC. Moreover, % extraction recovery ranged between 90.0-107.2 for SF and 93.1-108.0 for DC using the suggested method. The adopted chromatographic method was successfully applied to the therapeutic drug monitoring of SF and DC in healthy volunteers after the oral intake of one* Darvoni®* tablet.

## 1. Introduction

Infection due to hepatitis C virus (HCV) is a leading cause for severe chronic liver disease, which can result in progressive liver damage such as cirrhosis and hepatocellular carcinoma. Thus, it is considered to be a great worldwide health problem specifically in Egypt, which has the greatest prevalence of the epidemic problem of HCV in the world in accordance with the reported Egyptian Demographic Health Survey [EDHS] that had reached 14.7%. So prevention of HCV becomes a national priority [1].

The available treatment options for HCV infection until 2011 were restricted to ribavirin with pegylated interferon combination. This drug regimen has limited efficacy, especially in genotype 1 infected patients, and was also accompanied with dangerous side effects [2].

**Figure 1:**
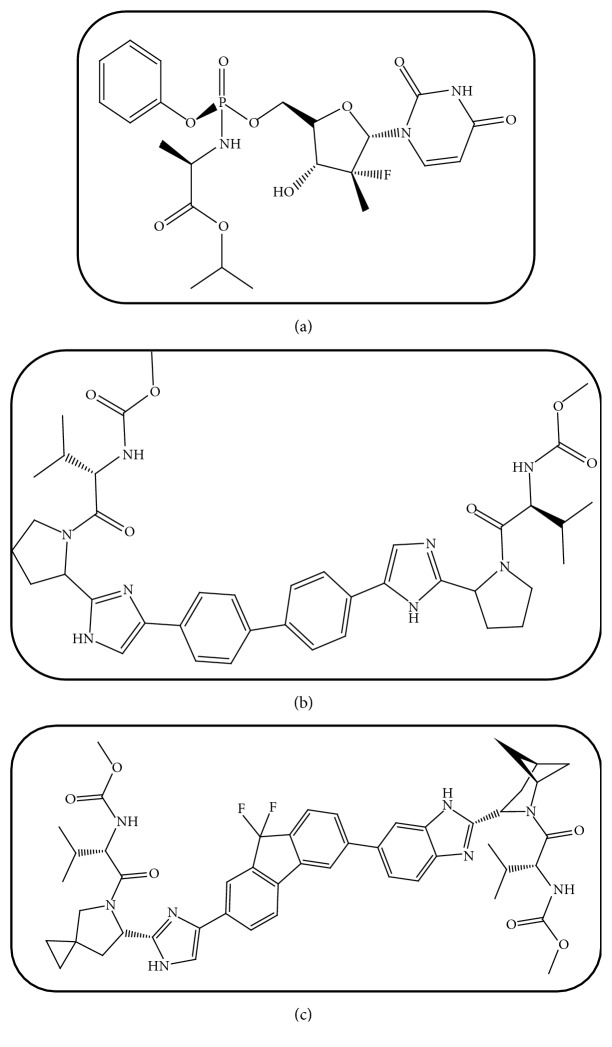
Chemical structure of SF (a), DC (b), and IS (c).

In 2014, the directly acting antivirals were introduced in the market as a new anti-HCV generation. The main goal of these new drug therapies is decreasing the incidence of possible side effects for HCV patients. These powerful drugs encompass Nonnucleoside Inhibitors (NNIs) and Nucleoside Inhibitors (NIs) of HCV RNA polymerase (NS5A/5B) and Protease Inhibitors (PIs). As shown in Figure 1(a), Sofosbuvir (SF) is the first released drug of the new generation and is an oral NI which is used alone or in combination with pegylated interferon *∝*/Ribavirin or with PIs as well as with NNIs (such as Ledipasvir or Daclatasvir) [3]. SF is a white to off-white crystalline solid having a molecular weight of 529.458 g/mol, with a solubility ≥ 2 mg/mL across the pH range of 2-7.7 at 37°C, and is slightly soluble in water. It has Log P value=1.62 and has two pKa values; pKa1= 9.38 (amide); pKa2 = 10.30[4].

Daclatasvir (DC) is the first discovered drug as HCV RNA polymerase NS5A replication complex inhibitor [5]. Its chemical structure is presented in Figure 1(b). DC is effective against genotypes 1 and 3. DC (molecular weight = 738.89 g/mol) is a white to yellow crystalline nonhygroscopic powder. It is freely soluble in water, dimethyl sulfoxide, and methanol; soluble in ethanol (95%); practically insoluble in dichloromethane, tetrahydrofuran, acetonitrile, acetone, and ethyl acetate. It has Log P value =4.67 and a pKa value = 11.15 [6]. DC has illustrated a good safety profile upon concomitant intake with SF [7]. The once-daily oral dose of anti-HCV concomitant drug therapy of SF + DC has shown high rates of sustained virologic response in patients infected with HCV genotype 1 or 3 and also has been accompanied with better therapeutic outcomes [8]. Ledipasvir (IS) is used as internal standard; its chemical structure is presented in Figure 1(c). Ledipasvir is practically insoluble (<0.1 mg/mL) across the pH range of 3.0-7.5 and is slightly soluble below pH 2.3 (1.1 mg/mL). It has a molecular weight of 889.018 g/mol, Log p value = 3.8. It has two pKa values: pka1 = 4.0 and pka2 = 5.0. [9]

The increase in number of therapeutic options available for patients suffering HCV infections introduces a great challenge in the selection and management of HCV treatment. In regard to this, Therapeutic Drug Monitoring (TDM) is considered to be an important tool to assess the efficacy of drug regimen, help the clinicians to adjust the drug dosage, optimize the therapy or switch the treatment regimen, and overcome adverse events or therapeutic failure [10, 11].

Due to the extensive use of the mentioned drugs in combination therapy, the therapeutic drug monitoring of concentrations of SF and DC concentrations in patients undergoing HCV therapy is very important and critical, especially in determining the treatment optimization and the potential of drug interaction.

Therefore, there were several reported methods for SF and/or DC determination in human plasma using HPLC-UV detection [12–14], micellar LC method [15], and LC-MS/MS [16–23].

To the best of our knowledge, the adopted bioanalytical approach in this work will be the solely UPLC-PDA technique first developed for the simultaneous quantification of SF and DC in human plasma with a clinical application to their therapeutic drug monitoring after the oral intake of a coformulated tablet containing SF+DC (Darvoni® recently manufactured by Beacon Pharmaceuticals Limited) instead of the commonly two tablets dosage regimen of each one alone (Sovaldi® + Daklinza®). In accordance with US Food and Drug Administration (FDA) guidelines [24], the adopted UPLC-DAD method in this work was successfully developed and carefully validated. Also a successful clinical application to the adopted method was also conducted through therapeutic drug monitoring process after the intake of Darvoni® tablet to three healthy volunteers.

## 2. Materials and Methods

### 2.1. Chemicals and Reagents

Drug standards for SF, DC, and Ledipasvir (IS) were kindly supplied by Memphis Co. for Pharmaceutical and Chemical Industries, Cairo, Egypt, with certified purity of 99.98 ± 0.421 for SF, 99.93 ± 0.231 for DC, and 99.87 ± 0.642 for Ledipasvir. Darvoni film-coated tablets (coformualted with 400 mg SF and 60 mg DC) were purchased from Beacon Pharmaceuticals Limited, Bangladesh. Drug-free human plasma was obtained from Kuwait Blood Bank, Al Jabriyah, Kuwait. HPLC grade acetonitrile and other used chemicals in the adopted method were of analytical grade and obtained from Sigma Aldrich, Dor-set, UK. “In house” HPLC grade water was prepared with a MilliQfilter purchased from Millipore, Watford, UK. Syringe membrane filters (13mm) were purchased from kinesis scientific expert, Cambridgeshire, UK. Nylon solvent filters (0.45 um) used for solvent filtration and Water 20-positions Extraction Manifold with SPE cartridges (Sep-Pak® Vac C18) used for sample preparation were purchased from Water Corporation, Milford, USA. SPE eluates were dried using DRI-BLOCK DB-3 evaporator which was purchased from Techne, Stone, UK.

### 2.2. Instrumentation and Chromatographic Conditions

Chromatographic separation was achieved using Waters® Acquity UPLC separation module with quaternary Solvent Manager (H-Class), online degasser with autosampler injector, and photodiode array detector coupled with Empower® software for data acquisition (Waters®, Milford, USA). Waters® Acquity UPLC BEH C_18_ column (2.1 mm × 50 mm, 1.7 *µ*m particle size) was used as the stationary phase for the development of the chromatographic separation, optimization, and method validation. An isocratic elution was conducted using a mobile phase system of 5 mM of ammonium formate in water and acetonitrile (60:40 v/v). The flow rate was set at 0.2 mL.min^−1^. Column temperature was adjusted at 45°C and samples were injected at 2 *µ*L injection volume and determined at a wavelength of 261 nm for SF and 318 nm for DC and IS.

### 2.3. Solutions

Due to the possible degradation of DC in solution at high-intensity UV and visible light as previously reported by [25], all the solutions and samples containing DC must be protected from daylight during its preparation, storage, and analysis. Two stock solutions for each of SF and DC were prepared in methanol as 1.0 mg.mL^−1^. Each stock standard solution was then further diluted with the same solvent to produce working standard solutions at the following concentration levels: 1.25, 2.5, 5.0, 10.0, 20.0, 80.0, 160.0, and 320.0 *µ*g.mL^−1^ for SF and 2.5, 5.0, 10.0, 20.0, 80.0, 160.0, 320.0, and 640.0 *µ*g.mL^−1^ for DC. Ledipasvir (IS) working standard solution was prepared as 50 *μ*g.mL^−1^ in methanol. Plasma calibration standards were freshly prepared at the following concentration levels: 25, 50, 100, 200, 400, 1600, 3200, and 6400 ng.mL^−1^ for SF and 50, 100, 200, 400, 1600, 3200, 6400, and 12800 ng.mL^−1^ for DC by dilution from their respective working solutions in control human plasma and subjected to subsequent analysis on the same day. The quality control (QC) samples used were subsequently prepared by further dilution of the working standard solutions in human plasma as presented in Table 1.

### 2.4. Preparation of Plasma Samples

Sample clean-up was conducted using the following SPE procedure: The cartridges were first preconditioned with acetonitrile (1 mL) then equilibrated by water (1 mL), in a positive pressure manifold. A volume of 450 *µ*L of acetonitrile and a volume of 50 *µ*L of IS were added to 500 *µ*L of the spiked human plasma samples, vortexed for one min approximately, and then centrifuged at room temperature for 10 min at 15,000 rpm to permit protein precipitation. Supernatants were then loaded onto SPE cartridges on a 20 position extraction manifold operated under positive vacuum. Then, the cartridges were washed with water (1 mL) and the analytes were successively eluted with acetonitrile. After that, the eluates collected from the previous step were evaporated under a N_2_ stream to dryness at 40°C. Then the dried residues were reconstituted with 125 *µ*L of mobile phase and centrifuged at 15,000 rpm for 7 min at room temperature and then filtered using syringe membrane filters (13mm) kinesis to be ready for injection and analysis into the UPLC system.

### 2.5. Method Validation

The assay validation was carried out in accordance with guidance for bioanalytical method validation recommended by the FDA [24]. Method development and validation for the adopted bioanalytical chromatographic method in plasma samples include the demonstration of (1) selectivity; (2) calibration curve, linearity, and sensitivity; (3) accuracy and precision; (4) recovery; and (5) stability of the analytes in the spiked plasma samples.

#### 2.5.1. Selectivity

The potential interferences from endogenous matrix components were investigated by evaluating ten different lots of human plasma as blank and at the LLOQ level of the spiked SF and DC. Drug-free plasma samples chromatograms were compared with those of the spiked plasma to ensure the absence of analytical interferences from endogenous substances present in plasma samples.

#### 2.5.2. Calibration Curve, Linearity, and Sensitivity

Eight-point calibration standard curves of SF and DC in plasma, ranging from 25 to 6400 ng.mL^−1^ and 50 to 12800 ng.mL^−1^, respectively, were prepared in triplicate for each run. The LLOQ is the lowest concentration in the standard calibration curve that back-calculates with good precision that does not exceed 20% of the CV and satisfactory accuracy which does not exceed 20% of the nominal concentration.

#### 2.5.3. Accuracy and Precision

Accuracy, intraday precision, and interday precision values were determined by the analysis of spiked human plasma samples with five different concentrations for each of SF and DC, corresponding to the LLOQ, low, medium, high QC samples, and ULOQ three times on the same day and on three separate days. Accuracy was expressed as the % of deviation between the nominal and measured concentration (% error). Precision was calculated as coefficient of variation % (CV).

#### 2.5.4. Recovery

The overall recovery of SF and DC from spiked human plasma was determined at the LLOQ, low, medium, high QC samples, and ULOQ. The ratio of the peak area response of extracted QC samples for SF or DC to that of the IS was compared to that of unextracted standards obtained by injecting the corresponding concentration of DC and IS in the mobile phase and analyzed in triplicate. The extraction recovery was computed as previously published [12, 26], using the ratio of the response and the assay concentration factor (500:125, since during SPE procedure 500 *µ*L spiked plasma samples was extracted, evaporated to dryness under a N_2_ stream at 40°C, and then reconstituted with 125 *µ*L of the mobile phase), and was expressed as % of the response of the calculated amount of SF or DC diluted in mobile phase and directly injected into the UPLC system, which corresponded to 100% recovery.

#### 2.5.5. Stability of the Analytes in the Spiked Plasma Samples

Stability tests were carried out under various conditions simulating those that a real human plasma samples may be subjected to during routine analysis. Stability studies of SF and DC in human plasma included the following:

(a) Freeze and thaw stability after three freeze-thaw cycles of stored plasma samples; (b) bench-top stability of plasma samples after storage at RT for 48 h; (c) long-term stability of plasma samples after storage at−80*◦*C for 6 months; stability of SF and DC stock solutions (d) kept in the refrigerator and (e) kept on bench; and processed sample stability (f) after the heating process for the frozen plasma samples (60°C for 60 min) in addition to (g) the stability of the dried extracts (after SPE procedure) at −20°C for 6 days and (h) the stability of the reconstituted extracts in the mobile phase after being kept at 4°C for 4 days in the autosampler.

For each of the previously mentioned conditions, three series of LLOQ, LQ, MQ, HQ, and ULOQ spiked plasma samples were analyzed. The SF and DC concentrations in the analyzed plasma samples were compared to freshly made QC samples. For all of the previously mentioned stability studies, SF and DC in plasma samples were considered as stable if the stability sample results were within 15% of nominal concentrations according to the FDA guidelines for bioanalytical method validation [24].

## 3. Results and Discussion

### 3.1. Chromatographic Separation Conditions and Sample Extraction Procedure

**Figure 2:**
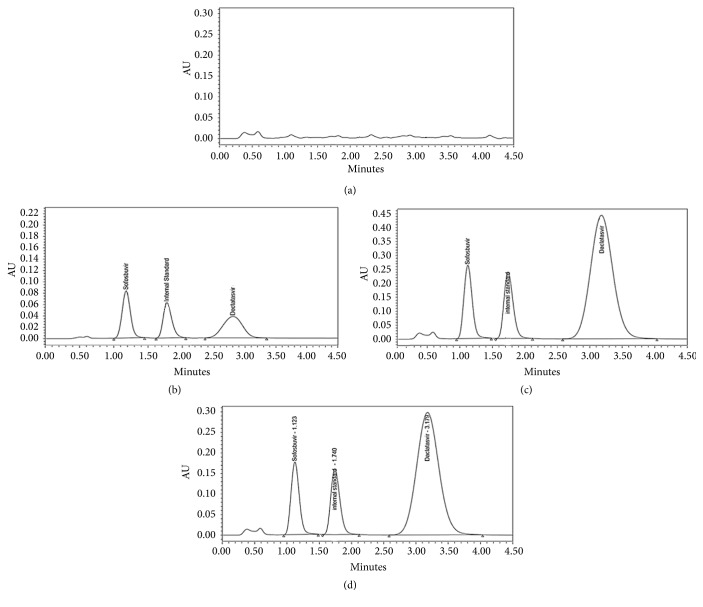
Typical UPLC chromatograms of (a) drug-free human plasma; (b) blank plasma spiked with SF and DC at LLOQ, (c) blank plasma spiked with SF and DC at ULOQ, and (d) plasma sample from a volunteer 0.5 h after administration of Darvoni® (400 mg of SF/60 mg of DC).

The chromatographic separation of SF and DC was carried out using different HPLC columns and various mobile phases. The proper chromatographic separation was achieved on Waters® Acquity UPLC BEH C_18_ column (2.1 × 50 mm, 1.7 *µ*m) using isocratic elution with a mobile phase of ammonium formate (pH 3.5; 5 mM) and acetonitrile (60:40 v/v) pumped at a flow rate 0.2 mL.min^−1^. According to these chromatographic conditions, SF was eluted at 1.123 min while DC was eluted at 3.179 min with a perfect separation from plasma endogenous peaks as presented in Figure 2. The total run time is considered to be short which allows faster analysis of multiple samples during routine work using simple isocratic elution. Figure 2 represents the chromatograms of (a) drug-free human plasma; (b) blank plasma spiked with SF and DC at LLOQ; (c) blank plasma spiked with SF and DC at ULOQ; and (d) plasma sample from a volunteer 0.5 h after administration of Darvoni® (400 mg of SF/60 mg of DC).

A satisfactory drug recovery in sample extraction is crucial for the simultaneous estimation of SF and DC in human plasma at low concentration levels. Various extraction procedures were tried such as liquid–liquid extraction with different solvents and protein precipitation techniques, but they gave very low drug recovery which lead to significant interference from plasma peaks background. Deproteinization using acetonitrile was carried out due to the fact that SF and DC are ~ 61-65% [27] and ~99% [28] bound to human plasma proteins, respectively, followed by a neat SPE procedure employing Water 20-positions Extraction Manifold with SPE cartridges (Sep-Pak® Vac C_18_) which was considered as a reliable and fast technique for determination of SF and DC in human plasma samples. This extraction strategy allowed the improvement of sample clean-up without lowering drug recovery leading to high and constant recovery of SF and DC from human plasma.

### 3.2. Method Validation

#### 3.2.1. Selectivity

Plasma sample extraction and chromatographic separation procedures were carried out to obtain a selective simultaneous determination for SF and DC. Various lots of blank human plasma from different sources were carefully evaluated for interference from endogenous matrix components. A typical chromatogram of blank human plasma is illustrated in Figure 2(a) which does not present any significant interfering endogenous peaks from human plasma at the retention times of SF, DC, or IS. The absence of any analytical interference was also assured using the peak purity checker system and matching with the library of the Empower® software.

#### 3.2.2. Calibration Curve, Linearity, and Sensitivity

Two calibration standard curves of SF and DC in plasma, ranging from 25 to 6400 ng.mL^−1^ and 50 to 12800 ng.mL^−1^, respectively, were prepared in triplicate for each run. The calibration plots showed linearity over the previously mentioned concentration ranges and the determination coefficient (r^2^) was not less than 0.998. The LLOQ is the lowest concentration in the standard calibration curve that back-calculates with good precision that does not exceed 20% of the CV and good accuracy within 20% of the nominal concentration. The LLOQ of SF and DC was 25 and 50 ng.mL^−1^, respectively. A typical chromatogram for a spiked plasma sample containing the LLOQ for both SF and DC is presented in Figure 2(b). This figure presents satisfactory sensitivity for the routine analysis of human plasma samples in clinical application of therapeutic drug monitoring.

#### 3.2.3. Accuracy and Precision

Data results for accuracy (expressed as % bias = [measured concentration – nominal concentration] / nominal concentration × 100) and precision (expressed as % CV) presented in Table 2 were less than 15% from the nominal concentrations and < 20% for LLOQ, according to FDA guidelines [24]. These results show that the adopted chromatographic method provides a high degree of accuracy and reproducibility.

#### 3.2.4. Extraction Recovery

The average of % extraction recovery for SF and DC in their respective concentration ranges varied from 90.0 to 107.2 % and 93.1 to 108.0 % for SF and DC, respectively, with % CV values ranging from 1.2 to 3.5 % and 1.1 to 3.6 % for SF and DC, respectively, as presented in Table 2. These data results demonstrate that the extraction procedure for human plasma samples produces clean extracts and good recovery to reach to satisfactory sensitivity for the analysis.

#### 3.2.5. Stability Studies

The stability of SF and DC in human plasma samples at five levels (LLOQ, LQ, MQ, HQ, and ULOQ) was investigated under the following conditions that the common and routine clinical samples are usually subjected to [29–31]: (a) after three freeze-thaw cycles (24 hours at −80°C to room temperature) on plasma samples spiked with SF and DC, the recovered concentrations of SF and DC were very similar to those of freshly spiked plasma samples. In addition, no decomposition products of SF or DC were detected during (b) short-term storage (48 h) of plasma samples at RT and (c) long-term storage at −80°C for 6 months as illustrated in Table 3 which confirms the high stability of both SF and DC in human plasma samples both at RT and at −80°C. Stock solution stability for SF and DC was also assessed. The stability of the standard solutions kept in refrigerator and those kept on bench was compared to freshly prepared standard solutions. It was found that solutions kept in (d) refrigerator are stable up to 10 days while those kept on (e) bench are stable for only 5 days. Moreover, the processed sample stability for SF and DC after the (f) heating process for the frozen plasma samples (60°C for 60 min) in addition to the stability of the (g) dried extracts (after SPE procedure) at −20°C for 6 days and in the processed plasma samples (h) reconstituted in the mobile phase after being kept at 4°C for 4 days in the autosampler was also tested. After the heating process for the frozen plasma samples (60°C for 60 min) and after 6 days storage of the dried extracts at −20°C or 4 days storage for the reconstituted extracts in the autosampler at 4°C, concentrations of the processed plasma samples were within ±15% of their nominal concentrations and < 20 % for LLOQ as presented in Table 3, indicating that both drugs are stable under the stated conditions to which routine plasma samples are subjected.

### 3.3. Application of the Adopted Method to the Plasma Samples of Healthy Volunteers

Concentrations of SF and DC were estimated using the adopted UPLC-PDA method in the plasma samples obtained from three healthy male volunteers after the oral intake of one Darvoni® tablet coformulated with 400 mg of SF and 60 mg of DC. To confirm the clinical application of the suggested method, a typical UPLC chromatogram of a plasma sample obtained from one of the volunteers after 0.5 h from the oral intake of one Darvoni® tablet is presented in Figure 2(d). The shown chromatogram assures satisfactory and good clinical application of the adopted chromatographic method during routine therapeutic drug monitoring. The good recovery of SF produced by the application of the proposed UPLC-PDA method makes no need to quantify the main metabolite of SF (GS-331007).

## 4. Conclusion

A rapid, accurate, precise, sensitive, and selective UPLC-PDA method was developed for simultaneous quantification of SF and DC in plasma samples has been adopted in this work for the first time. The developed method had been validated according to FDA guidelines for bioanalytical method validation. The method applicability was confirmed by the analysis of plasma samples of three healthy volunteers after the oral intake of coformulated Darvoni® tablet. So far, the previously published analytical methods developed for pharmacokinetic investigations of SF and DC were all based on costly LC-MS/MS equipment after the oral intake of two tablets for each drug, which decreased the feasibility of the routine analysis of SF and DC. The adopted UPLC-UV method is considered to be greatly applicable for the routine TDM of SF and DC in plasma at conventional clinical laboratories where LC-MS/MS equipment is not present. The suggested method will be suitable for standard clinical laboratories that do not possess LC-MS/MS equipment. With respect to large-scale pharmacokinetic analysis, the proposed method will be a satisfactory, simple, cheap, fast, and easier to set up alternative UPLC chromatographic method coupled with DAD. Stability studies of SF and DC under various common conditions to which both drugs may be subjected to during sample handling and analysis through routine TDM process illustrated that both drug concentrations remained approximately unchanged in plasma, in their stock solutions, and in processed plasma samples under different storage conditions. All in all, the adopted UPLC-DAD technique and the data results of the stability studies analyses could be valuable for dosing both drugs and appropriately dealing with their plasma samples both in routine clinical application and in TDM. This clinical application gave the opportunity to the optimization of the drug treatment which can improve the life quality and increase the therapy efficacy itself. It can lead also to a cost saving outcome, reducing side effects, and consequently a better clinical cost for patient's care.

## Figures and Tables

**Table 1:** Quality control samples for SF and DC.

Prepared QC samples (ng.mL^−1^)	SF	DC
LLOQ	25	50
QL	50	100
QM	400	1600
QH	3200	6400
ULOQ	6400	12800

**Table:** (a) Accuracy, intraday, and interday precision and % extraction recovery for SF and DC in their QC samples in human plasma (n=6)

Nominal Concentration (ng.mL^−1^)	**SF**				
	Average of the measured concentration (ng.ml^−1^)	Accuracy (% Bias)	Intra-day precision (% CV)	Inter-day precision (% CV)	% Extraction recovery ± %CV
25	24	-4.0	9.6	8.6	96.0 ± 1.2
50	45	-10.0	4.6	5.1	90.0 ± 3.5
400	425	6.3	5.2	9.3	106.3 ± 1.3
3200	3430	7.2	3.9	7.4	107.2 ± 2.8
6400	6123	-4.3	3.8	6.1	95.7 ± 1.5

Nominal Concentration (ng.mL^−1^)	**DC**				
	Average of the measured concentration (ng.ml^−1^)	Accuracy	Intra-day precision	Inter-day precision	% Extraction recovery ± %CV

50	54	8.0	6.5	3.7	108.0 ± 2.3
100	95	-5.0	9.2	9.1	95.0 ± 1.7
1600	1490	-6.9	4.7	7.5	93.1 ± 1.1
6400	6690	4.5	5.4	6.4	104.5 ± 2.0
12800	12357	-3.5	2.8	8.2	96.5 ± 3.6

**Table:** (b) Accuracy, intraday, and interday precision and % extraction recovery for SF and DC in their QC samples in human plasma (n=6)

Nominal Concentration (ng.mL^−1^)	**SF**				
	Average of the measured concentration (ng.ml^−1^)	Accuracy (% Bias)	Intra-day precision (% CV)	Inter-day precision (% CV)	% Extraction recovery ± %CV
25	24	-4.0	9.6	8.6	96.0 ± 1.2
50	45	-10.0	4.6	5.1	90.0 ± 3.5
400	425	6.3	5.2	9.3	106.3 ± 1.3
3200	3430	7.2	3.9	7.4	107.2 ± 2.8
6400	6123	-4.3	3.8	6.1	95.7 ± 1.5

Nominal Concentration (ng.mL^−1^)	**DC**				
	Average of the measured concentration (ng.ml^−1^)	Accuracy	Intra-day precision	Inter-day precision	% Extraction recovery ± %CV

50	54	8.0	6.5	3.7	108.0 ± 2.3
100	95	-5.0	9.2	9.1	95.0 ± 1.7
1600	1490	-6.9	4.7	7.5	93.1 ± 1.1
6400	6690	4.5	5.4	6.4	104.5 ± 2.0
12800	12357	-3.5	2.8	8.2	96.5 ± 3.6

**Table 3:** Stability of SF and DC in plasma samples under several storage conditions.

		Nominal Concentration (ng.ml^−1^)									
		**SF**					**DC**				
		25	50	400	3200	6400	50	100	1600	6400	12800
(a) After three freeze/thaw cycles	% Bias % CV	-2.9 5.4	5.2 6.5	-5.4 7.2	4.2 5.3	6.1 9.4	8.5 5.8	-3.5 1.5	-5.8 8.5	-3.1 3.2	-9.4 5.4

(b) short-term storage (48 h) at RT	% Bias % CV	4.1 1.1	4.1 6.5	7.3 5.5	-5.7 3.2	-4.8 5.2	5.1 6.6	6.4 7.3	9.2 1.5	8.4 2.4	-5.0 5.1

(c) long-term storage at −80°C for 6 months	% Bias % CV	3.5 2.1	-3.7 4.4	3.4 3.0	5.1 5.9	7.3 8.6	8.4 3.5	9.4 1.3	-2.0 7.0	-5.7 6.9	-6.3 3.5

(d) Standard solutions kept in refrigerator up to 10 days	% Bias % CV	-2.5 1.0	6.5 3.2	3.8 4.3	2.8 6.5	4.1 7.6	7.3 8.6	-5.7 5.7	-3.9 9.3	-2.8 1.0	1.7 1.3

(e) Solutions kept on bench are stable for 5 days	% Bias % CV	3.9 6.4	6.3 8.3	8.2 9.5	9.0 4.6	1.8 2.7	4.7 3.6	3.9 6.5	2.8 1.6	-3.7 4.9	-4.5 1.7

(f) Frozen plasma samples which undergo heating process (60°C for 60 min)	% Bias % CV	-6.7 5.4	-5.7 7.2	3.7 6.5	4.1 2.7	5.9 3.8	8.6 1.7	4.7 5.2	8.1 7.4	-9.4 6.9	-7.2 1.5

(g) Dried extracts (after SPE procedure) kept at −20°C for 6 days	% Bias % CV	9.5 5.3	4.7 6.2	5.8 8.3	-9.3 6.4	-6.3 9.3	-7.8 6.2	9.5 5.3	-5.2 8.1	-6.0 4.3	-3.7 3.8

(h) Reconstituted extracts in the mobile phase kept at 4°C for 4 days in the auto sampler	% Bias % CV	-5.7 4.6	-4.0 1.5	-5.2 4.6	4.9 4.1	8.1 5.3	7.2 9.4	7.6 8.3	6.1 1.7	8.5 3.2	5.3 8.4

## Data Availability

The data used to support the findings of this study are included within the article.
